# The degree of intubation difficulties and the frequency of complications in obese patients at the Hospital Emergency Department and the Intensive Care Unit: Case–control study

**DOI:** 10.1097/MD.0000000000005777

**Published:** 2016-12-30

**Authors:** Marcin Cierniak, Renata Sobczak, Dariusz Timler, Andrzej Wieczorek, Bartosz Borkowski, Tomasz Gaszyński

**Affiliations:** a Department of Emergency Medicine and Disaster Medicine, Medical University of Lodz; b University Laboratory of Teaching Medicine in Emergencies, Medical University of Lodz; c Department of Anesthesiology and Intensive Therapy, Medical University of Lodz, Lodz, Poland.

**Keywords:** intubation, obesity, FRONT score

## Abstract

The intubation difficulties in obese patients are not a new problem. They may result from an accumulation of fat in the oral cavity and cheeks. A thick tongue is also a significant factor. The literature reports that some tests to determine the intubation difficulties in obese people may be unreliable. The observed predictors of difficult intubation were the thyromental and sternomental distance and the intubation difficulty scale: FRONT score.

The aim of this study was to assess the degree of difficult intubation in obese patients by the parameters such as the thyromental and sternomental distance. The authors also tried to evaluate the frequency of the guidewire usage and the number of intubation attempts in obese patients in the research sample.

The study included the group of 153 patients intubated in prehospital conditions. The research was conducted in 3 clinical centers receiving patients from prehospital care. Among the members of the research sample, obese patients with body mass index >35 were selected and evaluated for various predictors of intubation difficulties. Quantitative analysis of differences in the incidence of the variables was assessed using the chi-squared test for *P* < 0.05. Analyses were performed in STATISTICA.Complications such as postintubation hematomas were more frequent in obese patients of the research sample. The frequency of the guidewire usage observed in that group was also higher. As anticipated by the adopted predictors, most of the obese patients were classified as difficult to intubate.

There is a correlation between the occurrence of injuries and the prevalence of obesity in the research sample and the same dependency has been demonstrated in the issue concerning the use of the guidewire. Although the majority of predictors indicated patients with intubation difficulties, many predictors could show falsely positive results. The greater amount of intubation attempts was observed in obese patients. Further studies devoted to explain those correlations would be needed.

## 1 Introduction

A difficult intubation is a condition in which performing a direct laryngoscopy, with a placement of the intubation tube below the laryngeal inlet, is problematic or impossible. The risk factors for difficult intubation include short and thick neck, a large tongue, impaired mobility of a neck and a jaw, or obesity.^[1]^ The main scope of interest in this article is obesity. In the obese (body mass index [BMI] > 35), the intubation difficulties result from the accumulation of fat in the oral cavity, cheeks, and a relatively thick tongue. It has been proved that although obesity itself does not necessarily predispose to difficult intubation, certain factors such as BMI > 35 and so called a thick neck defined as a neck circumference > 50 cm, or blurred edges of the mandible increase the likelihood of difficulties in laryngoscopy. These patients have usually a significantly shorter thyromental and sternomental distance.^[2]^ Despite a strong correlation between obesity and a Mallampati score or other predictors of a difficult intubation often found in the literature,^[3,4]^ we should consider that in the obese such test as a Mallampati score is not always reliable and partly because of that this scale was not used in our study.^[5,6]^ The purpose of this study was to assess the level of intubation difficulties in obese patients according to other indices of the difficult intubation, namely the thyromental and sternomental distance. The frequency of postintubation hematomas as complications was also rated. We also observed if the ratio of obese to nonobese patients increased with the higher rate of the FRONT score. Other factors considered in the analysis were the frequency of the guidewire usage and a number of intubation attempts in the obese from the research sample.

## 2 Materials and methods

The study was conducted in 2013/2014—jesli to zdanie zostaje to zrobiłabym jedno: The study was conducted in 2013/2014 and it included the group of 153 patients intubated in prehospital conditions. It was a case–control study performed in 3 clinical centers receiving patients from prehospital care. The study was approved by the Ethics Committee of the Medical University of Lodz in Poland (permission no. RNN/496/13KB) and written informed consent was given by each patient. To increase the reliability of the results, the study was carried out in 3 clinical centers: the Hospital Emergency Department of M. Kopernik Provincial Specialist Hospital in Łódź (33 patients), the Admission Room (50 patients), and the Intensive Care Unit (70 patients) of Barlicki University Hospital in Łódź. Differences in the number of patients in particular groups result from various incidence of patients meeting the inclusion criteria in a given period of time. Among these 153 patients, 48 obese patients were selected on the basis of BMI > 35. The study was concerned to assess the difficulties in intubation in the research sample according to the FRONT score and measure the thyromental and sternomental distance. The thyromental distance shorter than 6 cm indicates patients with a potential difficult intubation. It is worth noting that in case of this distance the results are often false positive. In case of the sternomental distance, the value below 12.5 cm with a head tilted back indicates a limit value for a difficult intubation. To achieve more effective assessment of the patient by using a particular scale, the video laryngoscope was used to visualize the airway. The visualization of the upper airway was performed by the Medical Doctor with PhD degree having at least the second degree of specialization in emergency medicine or anesthesiology and intensive care with a minimum of 15 years of work experience. The study was conducted with various types of video laryngoscopes (Table 1). The use of several types of the equipment was dictated by the subsequent comparative analysis concerning this subject. In addition to standard analyzes, the authors tried to present the dependencies among obesity and the occurrence of postintubation injuries, the FRONT score, and the intubation difficulty scale and thyromental and sternomental distance. We have also tried to check if there was a relationship between obesity and the guidewire usage or the number of intubation attempts in the obese from the research sample. Quantitative analysis of differences in the incidence of the variables was assessed using the chi-squared test for *P* < 0.05. All statistical analyses were performed in STATISTICA.

**Table 1:**
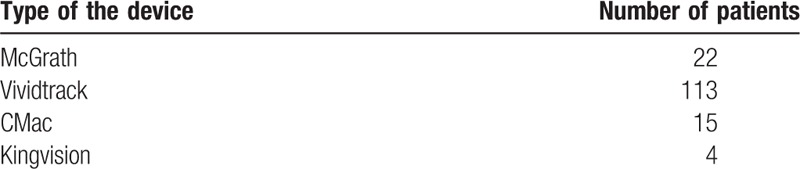
Patients examined with the particular devices.

## 3 Results

**Figure 1:**
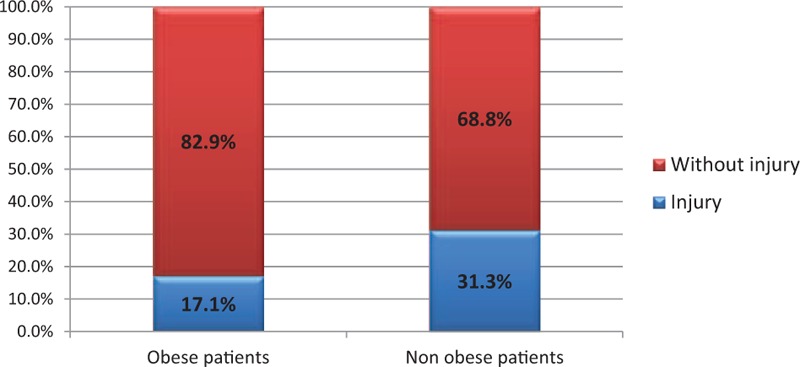
The incidence of injuries in the obese and nonobese patients.

**Figure 2:**
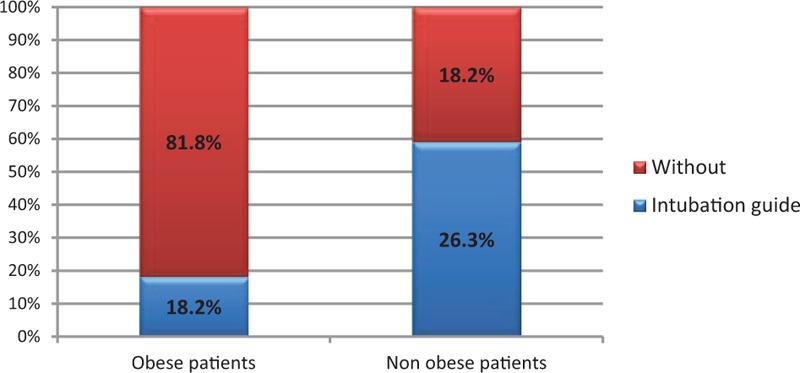
The percentage share of the obese patients for the particular values of the FRONT score.

**Figure 3:**
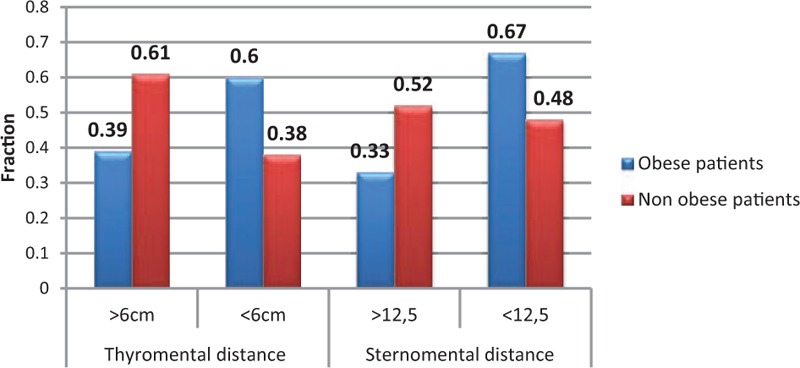
The thyromental and the sternomental distance in the obese and nonobese patients.

**Figure 4:**
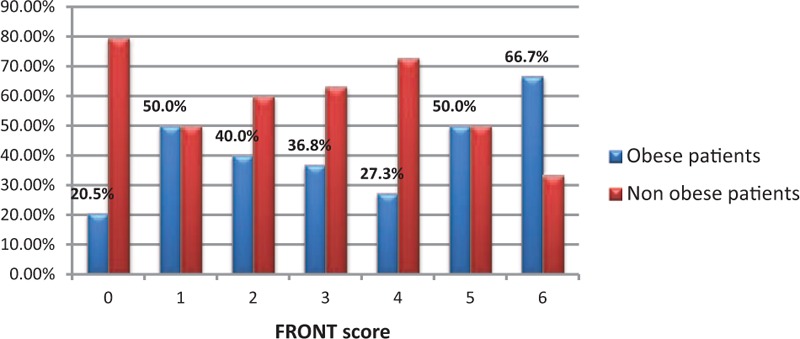
Guidewire usage in the obese and nonobese patients.

In the group of 153 patients, obesity was noted in 48 cases. There is a statistically significant relationship between obesity and the occurrence of postintubation hematomas. The chi-squared test, χ^2^ = 3.87, for critical values between 3.84 and 3.84 for *P* < 0.05. In the group of nonobese patients, 17.2% had some damages while in the group of the obese, the percentage of injuries almost doubled reaching 31.2% (Fig. 1). There was a weak relationship between obesity and the FRONT score. For almost every measured value detected on the scale FRONT (1.2–6), we noted a greater proportion of obese patients than in the group that scored 0 on this scale. The chi-squared test, χ^2^ = 13.09, for critical values from 12.59 to 12.59 for *P* < 0.05 (Fig. 2). Concerning the thyromental distance in the group of the obese, in case of the vast majority (Fr = 0.61), the measured distance was <6 cm and predisposed to difficult intubation. In the remaining part of the obese group (Fr = 0.39), the distance was ≥6 cm, and therefore based on this criterion, there were no expectations as for difficult intubation. The average thyromental distance in the obese was 5.11 ± 1.04 cm. Among nonobese patients from the research group, the proportions were almost reversed. Less than a half of them (Fr = 0.38) had the thyromental distance shorter than 6 cm, while the greater part of nonobese patients (Fr = 0.62) would be, in accordance with this criterion, easier to intubate due to the distance in excess of 6 cm. The average thyromental distance in nonobese patients was 5.77 ± 1.01 cm. There was a statistically significant relationship between the thyromental distance and obesity. The thyromental distance shorter than 6 cm was noted in only 30% of nonobese patients and in 52% of the obese. The chi-squared test, χ^2^ = 5.06, for critical values from 3.84 to 3.84 for *P* < 0.05. Regarding the sternomental distance in a group of obese patients in the vast majority of cases (Fr = 0.66), it was <12.5 cm and therefore predisposed to difficult intubation. The sternomental distance ≥12.5 cm was observed in less than a half of a group of obese patients (Fr = 0.34). The average sternomental distance in obese was 10.86 ± 1.44 cm. In nonobese patients from the research group, the proportions were almost reversed. In case of almost half of them (Fr = 0.46), the thyromental distance was shorter than 12.5 cm, while little more than a half of these patients (Fr = 0.54) would be, in accordance with this criterion, easier to intubate because of the distance exceeding 12.5 cm. The average thyromental distance in nonobese was 11.51 ± 1.61 cm. There was a statistically significant relationship between the sternomental distance and obesity. Among the nonobese, the sternomental distance shorter than 12.5 cm was noted in only 27% of people while among obese people the percentage was 55%. The chi-squared test, χ^2^ = 7.94, for critical values from 6.63 to 6.63 for *P* < 0.01 (Fig. 3). In 103 cases, we managed to contact a person who performed the intubation to ask about the number of intubation attempts. On the basis of these 103 patients, we found out that in the research sample, there was a statistically significant relationship between the number of intubation attempts and the occurrence of injuries. The chi-squared test, χ^2^ = 4.12, for critical values from 3.84 to 3.84 for *P* < 0.05. In the group of patients with single intubation attempt, the percentage of obese patients was 35%, while among those requiring 2 intubation attempts it increased to 58%. There was no statistically significant relationship between the size of the endotracheal tube and the incidence of obesity in the research sample. In the research sample, the size of the endotracheal tube ranged from 6 to 9. In case of the endotracheal tubes of sizes: 6.0, 6.5, and 7.0, the percentage of obese patients was <15% and regarding the tubes of larger sizes, the percentage of obese patients ranged from 25% to 28% (*P* > 0.7). As mentioned above, it was also difficult to contact the person who performed the intubation to ask whether the guidewire was used. It was possible only in 103 cases and sometimes we managed to reach this information basing on the amount of the waste equipment noted in the card filled in by the emergency medical services. In the group of these 103 patients, the guidewire was used in 20 cases. Among nonobese patients the guidewire was needed in 18.2% of cases, while in the obese it was used in 26.3% of patients (Fig. 4). Hence, there was a statistically significant relationship between obesity and the guidewire usage in the research sample. The chi-squared test, χ^2^ = 5.02, for critical values from 3.84 to 3.84 for *P* < 0.05.

## 4 Discussion

According to the worldwide literature, obesity itself is not a direct predictor and the factor influencing on peri-intubation complications, though the risk of complications in obese may be greater because obesity predisposes to difficult intubation.^[7]^ In our study, the occurrence of postintubation hematomas was perceived a complication and actually they were more frequent in obese patients from the research sample. Since we have shown that half of the cases in which the guidewire was used applied to obese patients, it may be related to the incidence of hematomas and that is also confirmed in the literature.^[8,9]^ The results of the research sample indicate that obesity of the examined patient is reflected in the exponential way in all considered indicators and predictors of difficult intubation. The authors did not distinguish between more or less obese patients, while in Holmberg's research such distinction showed the strongest relationship between obesity and the predictors of difficult intubation in the most obese patients and a much weaker association in less obese.^[10]^ Some authors postulate that every obese patient can be considered as potentially difficult to intubate, regardless of the indicators.^[11]^ Moreover, the literature reports that not all determinants are always good predictors.^[5]^ There are some findings reporting that for example not only Mallampati classification but also the thyromental distance can produce false-positive results. Although the majority of the obese was evaluated in accordance with the predictors as patients who are difficult to intubate, there was no statistically significant relationship between the considered factors, which in practice, should have occurred during difficult intubation in obese patient. We should also pay attention to the size of the endotracheal tube. We can assume that if we have the obese patient assessed as difficult to intubate, curve using different sizes and tubing should be guided downwards, but in this case it is the same as for people who were not rated as obese. On average, the same sizes of tubes were used in both groups. Considering the guidewire usage, we could assume that it would be used more often in the obese than in those with an appropriate BMI. However, even in this case, there was no statistically significant relationship. As it was mentioned above, the guidewire was used in 20 cases and exactly 10, so a half of those patients, was obese. We proved the association concerning the attempts of intubation. In case of patients who needed the second intubation attempt, there were more obese people (58% obese compared with 35% obese having single intubation attempt). Therefore, obese patients from the research sample might not have required the use of the guidewire or the smaller tube, but only more intubation attempts. It could be also the effect of the insufficient visibility while visualizing the laryngeal inlet. We might also consider stress and haste of prehospital conditions as other causes.^[12,13]^ It could be worth to examine it with using the video laryngoscopes that widen the field of view and enable for less attempts of intubation and shorten the time of intubation.^[14–17]^ Bearing in mind that obesity itself is not the direct predictor of complications,^[7,18]^ we should remember that in every case, the more intubation attempts the higher risk of complications.^[19,20]^

## 5 Limitations

The literature repeatedly demonstrated false dependencies among the predictors of difficult intubation, which frequently indicated both false-positive and false-negative results in the obese patients. This could distort the assessment in several of the patients from the research sample.

## 6 Conclusions

- There is a relationship between the incidence of injuries and obesity in the research sample. The same association was observed considering the guidewire usage.
- The relationship between the attempts of intubation and obesity in the research sample may lead to establishing the procedures limiting the intubation attempts and increasing the risk of complications.
- Further studies devoted to explain the causes of the relationships enumerated above, are needed.
